# 

**Published:** 1896-10

**Authors:** 


Vol XVII. OCTOBER, 1896. No. 10.

PUBLISHED MONTHLY AT $3 A YEAR. SINGLE COPIES, 30 CENTS.

### THE JOURNAL OF COMPARATIVE MEDICINE AND VETERINARY ARCHIVES

EDITED AND PUBLISHED BY
RUSH SHIPPEN HUIDEKOPER, Veterinarian (Alfort),
W. HORACE HOSKINS, D.V.S.,
H. D. GILL, V.S.

### UNITED STATES VETERINARY MEDICAL ASSOCIATION.

**Proceedings of the Thirty-third Annual Meeting held at Buffalo, N. Y., September, 1896.**

Minutes . . . . . . . . . . . . . . . . . . 1
Report of Committee on Army Legislation . . . . . . . . . . 23
Secretary's Report . . . . . . . . . . . . . . . 29
Reports of State Secretaries . . . . . . . . . . . . . 31

ALL COMMUNICATIONS AND PAYMENTS SHOULD BE ADDRESSED TO
**Dr. W. Horace Hoskins, Managing Editor, 3452 Ludlow St., Philadelphia, Pa.**
Copies may be had on order of all news-dealers. In London of Baillière, Tindall & Cox.

